# Heterocycle-containing tranylcypromine derivatives endowed with high anti-LSD1 activity

**DOI:** 10.1080/14756366.2022.2052869

**Published:** 2022-03-22

**Authors:** Rossella Fioravanti, Veronica Rodriguez, Jonatan Caroli, Ugo Chianese, Rosaria Benedetti, Elisabetta Di Bello, Beatrice Noce, Clemens Zwergel, Davide Corinti, Dolores Viña, Lucia Altucci, Andrea Mattevi, Sergio Valente, Antonello Mai

**Affiliations:** a Department of Drug Chemistry and Technologies, Sapienza University of Rome, Rome, Italy. Laboratory affiliated to Istituto Pasteur Italia-Fondazione Cenci Bolognetti; b Department of Biology and Biotechnology, University of Pavia, Pavia, Italy; c Department of Precision Medicine, University of Campania “Luigi Vanvitelli”, Naples, Italy; d Center for Research in Molecular Medicine and Chronic Disease (CIMUS), Department of Pharmacology, Pharmacy and Pharmaceutical Technology, Universidade de Santiago de Compostela, Santiago de Compostela, Spain; e Biogem Institute of Molecular and Genetic Biology, Ariano Irpino, Italy

**Keywords:** Epigenetics, histone demethylase, anticancer activity

## Abstract

As regioisomers/bioisosteres of **1a**, a 4-phenylbenzamide tranylcypromine (TCP) derivative previously disclosed by us, we report here the synthesis and biological evaluation of some (hetero)arylbenzoylamino TCP derivatives **1b**-**6**, in which the 4-phenyl moiety of **1a** was shifted at the benzamide C3 position or replaced by 2- or 3-furyl, 2- or 3-thienyl, or 4-pyridyl group, all at the benzamide C4 or C3 position. In anti-LSD1-CoREST assay, all the *meta* derivatives were more effective than the *para* analogues, with the *meta* thienyl analogs **4b** and **5b** being the most potent (IC_50_ values = 0.015 and 0.005 μM) and the most selective over MAO-B (selectivity indexes: 24.4 and 164). When tested in U937 AML and prostate cancer LNCaP cells, selected compounds **1a,b**, **2b**, **3b**, **4b**, and **5a,b** displayed cell growth arrest mainly in LNCaP cells. Western blot analyses showed increased levels of H3K4me2 and/or H3K9me2 confirming the involvement of LSD1 inhibition in these assays.

## Graphical Abstract

**Figure:**
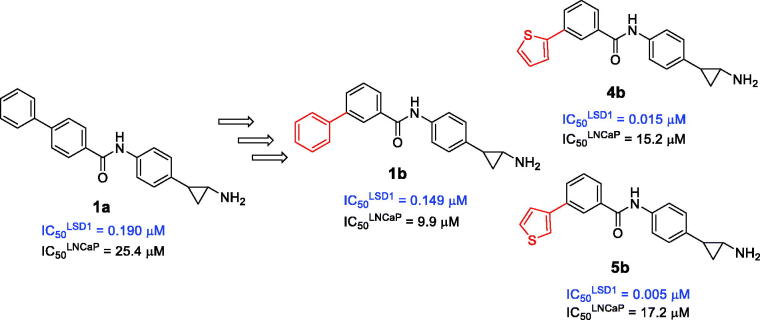

## 1. Introduction

Protein post-translational modifications (PTMs), such as ac(et)ylation, methylation, and phosphorylation, play an important role in regulating many protein functions. Among such PTMs, reversible methylation/demethylation of lysine residues is typically involved in regulating the chromatin environment and eukaryotic gene expression^1^^,^^2^.

In mammals there are two families of lysine demethylases (KDMs): the lysine-specific demethylases (LSDs, including LSD1 and −2, also called KDM1A and -B) and the jumonij-containing demethylases, called KDM2-8^2^. LSD1 is a flavoenzyme that specifically removes the methyl groups of H3K4me1/2 and H3K9me1/2 in complexes with various chromatin-associated factors including CoREST^3–7^. LSD2 also demethylates histone H3, mainly to mono- and dimethylated Lys4, although its activity is weaker than that of LSD1 *in vitro*^8^.

LSD1 plays a vital role in a broad spectrum of biological processes, including embryonic development, stem cell maintenance and differentiation, and is also a key player in oncogenic processes, including cancer cell growth and metastasis. LSD1 has been reported to be overexpressed in a variety of cancers, and its inactivation or downregulation of its expression inhibits the development of cancer cells^2^^,^^9^. LSD1 regulates cellular signalling pathways in various cancerous entities such as breast cancer, where it interacts in a multiprotein complex containing deacetylase (NuRD)^10^. In prostate cancer, high levels of LSD1 are associated with cancer progression and metastasis and therefore LSD1 levels could be a useful biomarker^11^^,^^12^. LSD1 and LSD2 share a similar catalytic domain (45% sequence identity) that is structurally homologous with the amine oxidases, a class of flavin-dependent enzymes that act on biogenic amines^5^^,^^13^. Among these proteins, human monoamine oxidases (MAOs) A and B have been the subject of more than 50 years of research that has led to the development of a multitude of inhibitors including antidepressant and antiparkinson drugs^14^. Their similarity in the catalytic and structural properties prompted the investigation of anti-MAO drugs as potential LSD1 inhibitors^15^. Among them, tranylcypromine (TCP) is one of the most reliable molecules able to inhibit LSD1 irreversibly via a covalent interaction with the FAD cofactor and has been widely used as a fragment to create valuable LSD1 inhibitors^16^.

**Figure 1.**
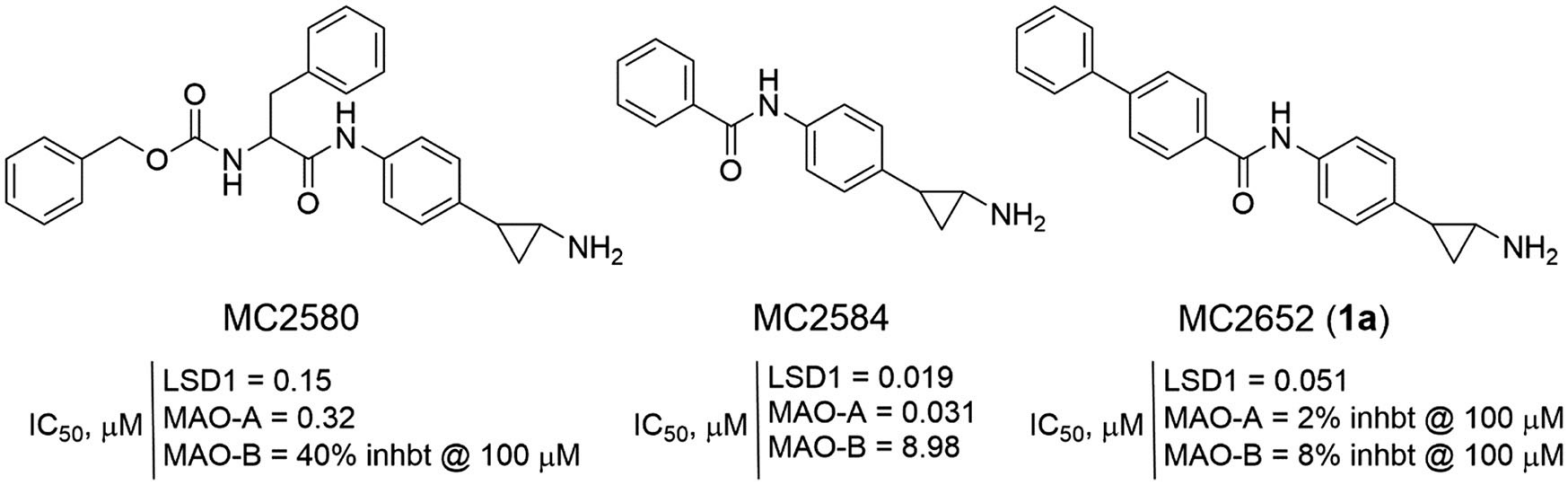
TCP-based LSD1 inhibitors disclosed by us.

In the past, we have described numerous TCP-containing compounds as covalent LSD1 inhibitors^17–26^, and among them, MC2580^17^^,^^26^^,^^27^ and MC2584^17^^,^^18^ (Figure 1) were the most effective prototypes at enzyme level. MC2580 showed a quite good selectivity for LSD1 over MAO-B (not MAO-A), and it strongly enhanced the efficacy of retinoic acid on growth and differentiation of acute promyelocytic leukaemia (APL) cells, including primary murine APL blasts^17^. The two enantiomers of MC2584 displayed at 0.25 μM strong (64%, 1 *R,*2*S*, and 83.4%, 1*S*,2*R*) inhibition on colony formation in the same murine APL blasts^18^. The introduction of a phenyl ring at the *para* position of the MC2584 benzoylamino moiety led to MC2652 (**1a**) (Figure 1), less potent in LSD1 inhibition but more selective towards MAOs, and able to arrest MV4-11 acute myeloid leukaemia (AML) and NB4 APL cell growth with IC_50_ values at low, single-digit micromolar levels^25^.

**Figure 2.**
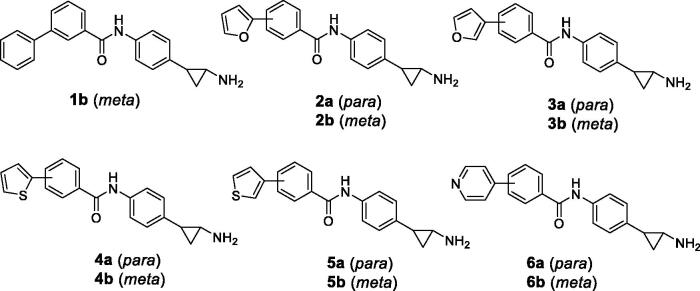
Novel TCP-based analogs as LSD1 inhibitors **1 b**-**6**.

Based on these data, we designed and synthesised new analogs of **1a** as potential inhibitors of LSD1, by shifting the 4-phenyl ring from the 4- to the 3-position of the benzoylamino moiety (**1b**) (Figure 2) and replacing this 4- or 3-phenyl ring with different heterocyclic moieties such as furan, thiophene, and pyridine (**2**–**6**) (Figure 2), aiming to improve their potency and LSD1 selectivity. Selected compounds were tested against U937 AML and prostate LNCaP cancer cells to assess their antiproliferative potential.

## 2. Materials and methods

### 2.1. Chemistry

Melting points were determined on a Buchi 530 melting point apparatus and are uncorrected. ^1^H and ^13 ^C NMR spectra were recorded at 400 MHz and 100 MHz, respectively, on a Bruker AC 400 spectrometer; chemical shifts are reported in *δ* (ppm) units relative to the internal reference tetramethylsilane (Me_4_Si). EIMS spectra were recorded with a Thermo MSQ Plus spectrometer; only molecular ions (M^+^) and base peaks are given. All the compounds were routinely checked by TLC, ^1^H NMR and ^13 ^C NMR spectra. TLC was performed on aluminum-backed silica gel plates (Merck DC, Alufolien Kieselgel 60 F_254_) with spots visualised by UV light. All solvents were reagent grade and, when necessary, were purified and dried by standard methods. The concentration of solutions after reactions and extractions involved the use of a rotary evaporator operating at reduced pressure of ca. 20 Torr. Organic solutions were dried over anhydrous sodium sulphate. Elemental analysis has been used to determine the purity of the described compounds, that is >95%. Analytical results are within ± 0.40% of the theoretical values. All chemicals were purchased from Sigma Aldrich, Milan (Italy), or from TCI Europe, Zwjindrecht (Belgium), and were of the highest purity.

### 2.2. *General* procedure for the synthesis of the tert-butyl (2-(4-(4- or 3-(phenyl/heteroaryl)benzamido)phenyl)cyclopropyl)carbamates 14–19 *example:*

**tert*-butyl (2-(4-([1,1′-biphenyl]-3-carboxamido)phenyl)cyclopropyl)carbamate (14b).*** Triethylamine (1.05 mmol, 0.15 ml) and benzotriazol-1-yloxytripyrrolidino-phosphonium hexafluorophosphate (PyBOP) (0.24 mmol, 0.126 g) were added to a solution of [1,1′-biphenyl]-3-carboxylic acid (0.20 mmol, 0.04 g) in anhydrous *N*,*N*-dimethylformamide (3 ml) under a nitrogen atmosphere. The resulting mixture was stirred for 45 min at room temperature followed by the addition of *tert*-butyl (2-(4-aminophenyl)cyclopropyl)carbamate **13**^17^ (0.24 mmol, 0.06 g) under a nitrogen atmosphere, and the reaction was stirred overnight. The reaction was quenched with brine (50 ml) and the precipitated solid was filtered off and washed with water (3 × 5 ml). The residue was purified by chromatographic column on silica gel eluting with ethyl acetate/chloroform 1:3 to afford the pure **14b**. Chemical and physical data: m.p., 212–214 °C; yield, 64%. ^1^H NMR (DMSO-d_6_, 400 MHz, *δ*; ppm) *δ* 1.06–1.09 (m, 2H, CH_2_ cyclopropane), 1.39 (s, 9H, (CH_3_)_3_), 1.86–1.89 (m, 1H, CHNH_2_ cyclopropane), 2.62 (bs, 1H, PhCH cyclopropane), 7.09– 7.11 (d, 2H, benzene protons, *J* = 8 Hz), 7.24 (bs, 1H, benzene protons), 7.42–7.44 (t, 1H, benzene proton, *J* = 8 Hz), 7.49–7.46 (t, 2H, benzene protons, *J* = 12 Hz), 7.61–7.64 (t, 1H, benzene proton, *J* = 12 Hz), 7.67–7.69 (d, 2H, benzene protons, *J* = 8 Hz), 7.71–7.73 (d, 2H, benzene protons, *J* = 8 Hz), 7.87–7.89 (m, 1H, benzene proton), 7.93–7.95 (d, 1H, benzene proton, *J* = 8 Hz), 8.22 (bs, 1H, CONH), 10.28 (bs, 1H, CONH), ppm; ^13 ^C (100 MHz, DMSO-d_6_) *δ* 166.9, 155.44, 140.6, 139.7, 139.1, 137.9, 133.4, 130.9, 130.2, 129.1, 128.1, 127.9, 127.7 (3 C), 127.3, 127.1 (3 C), 121.2 (3 C), 79.7, 33.8, 28.3, 26.3, 18.1; MS (EI) *m/z*: 428.21 [M]^+^.

**tert-*Butyl (2-(4-(4-(furan-2-yl)benzamido)phenyl)cyclopropyl)carbamate (15a)*.** Chemical and physical data: m.p., 223–225 °C; yield, 49%. ^1^H NMR (CDCl_3_, 400 MHz, *δ*; ppm) *δ* 1.09–1.12 (m, 2H, CH_2_ cyclopropane), 1.39 (s, 9H, (CH_3_)_3_), 1.98 (bs, 1H, CHNH_2_ cyclopropane), 2.63 (bs, 1H, PhCH cyclopropane), 4.81 (bs, 1H, CONH), 6.45–6.46 (m, 1H, furan proton), 6.71–6.72 (d, 1H, furan proton, *J =* 4 Hz), 7.07–7.09 (d, 2H, benzene protons, *J =* 8 Hz), 7.46–7.49 (m, 3H, furan and benzene protons), 7.69–7.71 (d, 2H, benzene protons, *J =* 8 Hz), 7.76 (bs, 1H, CONH), 7.81–7.83 (d, 2H, benzene protons, *J =* 8 Hz) ppm; ^13 ^C (100 MHz, DMSO-d_6_) *δ* 168.5, 155.4, 153.2, 142.4 (2 C), 139.1, 137.9, 137.2, 134.2, 128.9 (2 C), 127.3 (2 C), 125.1 (2 C), 121.2 (2 C), 111.0, 107.7, 79.7 (2 C), 33.8, 28.3, 26.3, 18.1; MS (EI) *m/z*: 418.19 [M]^+^.

**tert-*Butyl (2-(4-(3-(furan-2-yl)benzamido)phenyl)cyclopropyl)carbamate (15b).*** Chemical and physical data: m.p., 197–200 °C; yield, 77%. ^1^H NMR (CDCl_3_, 400 MHz, *δ*; ppm) *δ* 1.28–1.29 (m, 2H, CH_2_ cyclopropane), 1.51 (s, 9H, (CH_3_)_3_), 1.96–198 (m, 1H, CHNH_2_ cyclopropane), 2.64 (bs, 1H, PhCH cyclopropane), 4.85 (bs, 1H, CONH), 6.43–6.45 (d, 1H, furan proton, *J =* 8 Hz), 6.69–6.71 (d, 1H, furan proton, *J =* 8 Hz), 7.10–7.12 (d, 2H, benzene protons, *J =* 8 Hz), 7.44–7.45 (m, 2H, benzene protons), 7.48–7.50 (d, 2H, benzene protons, *J =* 8 Hz), 7.65–7.67 (d, 1H, furan proton, *J =* 8 Hz), 7.75–7.79 (m, 2H, benzene protons), 8.08 (bs, 1H, CONH) ppm; ^13 ^C (100 MHz, DMSO-d_6_) *δ* 166.7, 155.4, 154.2, 142.3 (2 C), 139.1, 137.9, 133.3, 130.7 (2 C), 128.3, 128.1, 127.3 (2 C), 126.5, 121.2 (2 C), 111.5, 107.7, 79.7 (2 C), 33.8, 28.3, 26.3, 18.1; MS (EI) *m/z*: 418.19 [M]^+^.

**tert-*Butyl (2-(4-(4-(furan-3-yl)benzamido)phenyl)cyclopropyl)carbamate (16a).*** Chemical and physical data: m.p., 232–234 °C; yield, 49%. ^1^H NMR (CDCl_3_, 400 MHz, *δ*; ppm) *δ* 1.08–1.09 (m, 2H, CH_2_ cyclopropane), 1.39 (s, 9H, (CH_3_)_3_), 1.97–1.98 (m, 1H, CHNH_2_ cyclopropane), 2.63 (bs, 1H, PhCH cyclopropane), 4.80 (bs, 1H, CONH), 6.68 (bs, 1H, furan proton), 7.07–7.09 (d, 2H, benzene protons, *J =* 8 Hz), 7.43–7.46 (m, 3H, furan and benzene protons), 7.51–7.53 (d, 2H, benzene protons, *J =* 8 Hz), 7.71–7.73 (m, 1H, furan protons), 7.75 (bs, 1H, CONH), 7.79–7.81 (d, 2H, benzene protons, *J =* 8 Hz) ppm; ^13 ^C (100 MHz, DMSO-d_6_) *δ* 168.2, 155.5, 143.0, 140.77, 139.1 (2 C), 138.4, 137.8, 136.6, 130.1 (2 C), 127.3 (2 C), 127.2, 126.6 (2 C), 121.1 (2 C), 108.8, 79.7 (2 C), 33.9, 28.3, 26.3, 18.5; MS (EI) *m/z*: 418.19 [M]^+^.

**tert-*Butyl (2-(4-(3-(furan-3-yl)benzamido)phenyl)cyclopropyl)carbamate (16b).*** Chemical and physical data: m.p., 196–198 °C; yield, 41%. ^1^H NMR (CDCl_3_, 400 MHz, *δ*; ppm) *δ* 1.08–1.10 (m, 2H, CH_2_ cyclopropane), 1.39 (s, 9H, (CH_3_)_3_), 1.97–1.99 (m, 1H, CHNH_2_ cyclopropane), 2.64 (bs, 1H, PhCH cyclopropane), 4.80 (bs, 1H, CONH), 6.69 (bs, 1H, furan proton), 7.08–7.10 (d, 2H, benzene protons, *J =* 8 Hz), 7.41–7.47 (m, 4H, furan and benzene protons), 7.57–7.62 (m, 2H, benzene protons), 7.74 (bs, 2H, furan and benzene protons), 7.92 (bs, 1H, CONH) ppm; ^13 ^C (100 MHz, DMSO-d_6_) *δ* 166.4, 155.5, 141.9, 140.7, 139.1, 138.4, 133.9 (2 C), 133.9, 130.3, 129.9, 128.5, 127.4, 127.3 (2 C), 126.8 (2 C), 121.1 (2 C), 109.5, 79.7, 33.9, 28.3, 26.3, 18.5; MS (EI) *m/z*: 418.19 [M]^+^.

**tert-*Butyl (2-(4-(4-(thiophen-2-yl)benzamido)phenyl)cyclopropyl)carbamate (17a).*** Chemical and physical data: m.p., 226–228 °C; yield, 35%. ^1^H NMR (CDCl_3_, 400 MHz, *δ*; ppm) *δ* 1.18–1.21 (m, 2H, CH_2_ cyclopropane), 1.39 (s, 9H, (CH_3_)_3_), 1.95–1.97 (m, 1H, CHNH_2_ cyclopropane), 2.64 (bs, 1H, PhCH cyclopropane), 4.78 (bs, 1H, CONH), 7.05–7.09 (m, 3H, thiophene protons), 7.29–7.30 (m, 1H, benzene proton), 7.34– 7.36 (m, 1H, benzene protons), 7.47–7.49 (d, 2H, benzene protons, *J =* 8 Hz), 7.64–7.66 (d, 2H, benzene protons, *J =* 8 Hz), 7.70 (bs, 1H, CONH), 7.79–7.82 (d, 2H, benzene protons, *J =* 12 Hz), ppm; ^13 ^C (100 MHz, DMSO-d_6_) *δ* 168.2, 155.5 (2 C), 143.0, 139.2, 139.1, 138.4, 136.9, 128.6 (2 C), 127.9, 127.3 (2 C), 126.9, 126.8 (2 C), 124.7, 121.1 (2 C), 79.7, 33.9 (2 C), 28.3, 26.3, 18.5; MS (EI) *m/z*: 434.17 [M]^+^.

**tert-*Butyl (2-(4-(3-(thiophen-2-yl)benzamido)phenyl)cyclopropyl)carbamate (17b).*** Chemical and physical data: m.p., 189–191 °C; yield, 55%. ^1^H NMR (CDCl_3_, 400 MHz, *δ*; ppm) *δ* 1.12–1.15 (m, 2H, CH_2_ cyclopropane), 1.45 (s, 9H, (CH_3_)_3_), 2.02–2.04 (m, 1H, CHNH_2_ cyclopropane), 2.70 (bs, 1H, PhCH cyclopropane), 4.82 (bs, 1H, CONH), 7.09–7.12 (t, 1H, thiophene proton, *J =* 12 Hz), 7.15–7.17 (d, 2H, benzene proton, *J =* 8 Hz), 7.32– 7.34 (d, 1H, thiophene proton, *J =* 8 Hz), 7.39–7.40 (d, 1H, benzene proton, *J =* 4 Hz), 7.48–7.51 (m, 1H, thiophene proton), 7.53–7.56 (d, 2H, benzene protons, *J =* 12 Hz), 7.70–7.78 (m, 3H, benzene protons), 8.09 (bs, 1H, CONH) ppm; ^13 ^C (100 MHz, DMSO-d_6_) *δ* 165.9, 155.5 (2 C), 141.70, 139.1, 138.4, 135.9 (2 C), 132.8, 130.3, 128.5, 128.3, 128.1, 127.8, 127.3 (2 C), 126.5, 124.4, 121.1 (2 C), 79.7, 33.9, 28.3, 26.3, 18.5; MS (EI) *m/z*: 434.17 [M]^+^.

**tert-*Butyl (2-(4-(4-(thiophen-3-yl)benzamido)phenyl)cyclopropyl)carbamate (18a).*** Chemical and physical data: m.p., > 287 °C; yield, 40%. ^1^H NMR (CDCl_3_, 400 MHz, *δ*; ppm) *δ* 1.09–1.11 (m, 2H, CH_2_ cyclopropane), 1.39 (s, 9H, (CH_3_)_3_), 1.97–1.98 (m, 1H, CHNH_2_ cyclopropane), 2.64 (bs, 1H, PhCH cyclopropane), 4.78 (bs, 1H, CONH), 7.08–7.10 (m, 2H, thiophene protons), 7.37 (bs, 2H, benzene protons), 7.48–7.51 (m, 3H, thiophene and benzene protons), 7.61–7.63 (d, 2H, benzene protons, *J =* 8 Hz), 7.71 (bs, 1H, CONH), 7.81–7.83 (d, 2H, benzene protons, *J =* 8 Hz) ppm; ^13 ^C (100 MHz, DMSO-d_6_) *δ* 168.5, 155.4, 141.7, 140.5, 139.1, 137.9, 136.8, 129.7 (3 C), 127.7, 127.3, 127.2 (3 C), 127.1, 122.4, 121.2 (3 C), 79.7, 33.8, 28.3, 26.3, 18.1; MS (EI) *m/z*: 434.17 [M]^+^.

**tert-*Butyl (2-(4-(3-(thiophen-3-yl)benzamido)phenyl)cyclopropyl)carbamate (18b).*** Chemical and physica data: m.p., 214–216 °C; yield, 56%. ^1^H NMR (CDCl_3_, 400 MHz, *δ*; ppm) *δ* 1.09–1.11 (m, 2H, CH_2_ cyclopropane), 1.39 (s, 9H, (CH_3_)_3_), 1.98–2.02 (m, 1H, CHNH_2_ cyclopropane), 2.64 (bs, 1H, PhCH cyclopropane), 4.81 (bs, 1H, CONH), 7.07–7.09 (d, 2H, benzene protons, *J =* 8 Hz), 7.36–7.38 (m, 2H, thiophene and benzene protons), 7.41–7.47 (m, 4H, thiophene and benzene protons), 7.65–7.67 (t, 2H, thiophene and benzene protons, *J =* 8 Hz), 7.78 (bs, 1H, benzene protons), 8.03 (bs, 1H, CONH) ppm; ^13 ^C (100 MHz, DMSO-d_6_) *δ* 166.9, 155.4, 140.8, 139.1, 138.6, 137.9, 133.6, 130.3, 130.2, 128.4, 127.4, 127.3 (3 C), 127.1, 127.1, 122.6, 121.2 (3 C), 79.7, 33.8, 28.3, 26.3, 18.1; MS (EI) *m/z*: 434.17 [M]^+^.

**tert-*Butyl (2-(4-(4-(pyridin-4-yl)benzamido)phenyl)cyclopropyl)carbamate (19a).*** Chemical and physical data: m.p., 198–200 °C; yield, 40%. ^1^H NMR (CDCl_3_, 400 MHz, *δ*; ppm) *δ* 1.09–1.11 (m, 2H, CH_2_ cyclopropane), 1.44 (s, 9H, (CH_3_)_3_), 1.98–1.99 (m, 1H, CHNH_2_ cyclopropane), 2.64 (bs, 1H, PhCH cyclopropane), 4.81 (bs, 1H, CONH), 7.09–7.11 (m, 2H, benzene protons), 7.47–7.49 (m, 4H, pyridine and benzene protons), 7.67–7.69 (d, 2H, benzene protons, *J =* 8 Hz), 7.80 (bs, 1H, CONH), 7.91–7.93 (d, 2H, benzene protons, *J =* 8 Hz), 8.63–8.65 (m, 2H, pyridine protons) ppm; ^13 ^C (100 MHz, DMSO-d_6_) *δ* 168.5, 155.4, 148.2 (2 C), 147.5, 141.8, 139.1, 137.9, 136.8 (2 C), 129.6 (2 C), 128.4 (2 C), 127.3 (2 C), 121.6 (2 C), 121.2 (2 C), 79.7, 33.8, 28.3, 26.3, 18.1 (2 C); MS (EI) *m/z*: 429.21 [M]^+^.

**tert-*Butyl (2-(4-(3-(pyridin-4-yl)benzamido)phenyl)cyclopropyl)carbamate (19b).*** Chemical and physical data: m.p., 158–160 °C; yield, 70%. ^1^H NMR (CDCl_3_, 400 MHz, *δ*; ppm) *δ* 1.15–1.16 (m, 2H, CH_2_ cyclopropane), 1.44 (s, 9H, (CH_3_)_3_), 2.03–2.04 (m, 1H, CHNH_2_ cyclopropane), 2.63 (bs, 1H, PhCH cyclopropane), 5.12 (bs, 1H, CONH), 7.13–7.15 (m, 2H, benzene protons), 7.55–7.62 (m, 6H, pyridine and benzene protons), 7.79–7.81 (d, 1H, benzene protons, *J =* 8 Hz), 7.93–7.95 (d, 1H, benzene protons, *J =* 8 Hz), 8.19 (bs, 1H, CONH), 8.64 (bs, 2H, pyridine protons) ppm; ^13 ^C (100 MHz, DMSO-d_6_) *δ* 166.9, 155.4, 150.2 (2 C), 146.2, 139.3, 139.1, 137.9, 133.4, 131.5, 130.2, 128.1, 128.0, 127.3 (2 C), 121.7 (3 C), 121.2 (3 C), 79.7, 33.8, 28.3, 26.3, 18.1; MS (EI) *m/z*: 429.21 [M]^+^.

### 2.3. *General* procedure for the synthesis of the *N*-(4-(2-aminocyclopropyl)phenyl)-4- or 3-(phenyl/heteroaryl)benzamides 1–6 as hydrochloride salts *example*

**N*-(4-(2-Aminocyclopropyl)phenyl)-[1,1′-biphenyl]-3-carboxamide hydrochloride (1b).*** A solution of *tert*-butyl (2-(4-([1,1′-biphenyl]-3-carboxamido)phenyl)cyclopropyl)carbamate (**14b**) (0.12 mmol, 0.05 g) in dry THF (8 ml) was treated with 4 N HCl in dioxane (1.20 mmol, 0.3 ml) at 0 °C, and the resulting reaction mixture was left under stirring at rt for 24 h. After the end of the reaction, the resulting precipitate was isolated by filtration and washed with Et_2_O to give the pure **1b** as hydrochloride salt. Chemical and physical data: m.p., >250 °C (methanol); yield, 57%. ^1^H NMR (DMSO-d_6_, 400 MHz, *δ*; ppm) *δ* 1.19–1.21 (m, 1H, CHH cyclopropane), 1.21–1.28 (m, 1H, CHH cyclopropane), 2.31–2.39 (m, 1H, CHNH_2_ cyclopropane), 2.77–2.81 (m, 1H, PhCH cyclopropane), 7.16–7.18 (d, 2H, benzene protons, *J =* 8 Hz), 7.41–7.45 (t, 1H, benzene proton, *J =* 16 Hz), 7.50–7.55 (t, 2H, benzene protons, *J =* 20 Hz), 7.60–7.65 (t, 1H, benzene protons, *J =* 20 Hz), 7.72–7.80 (m, 4H, benzene protons), 7.87–7.90 (d, 1H, benzene protons, *J =* 12 Hz), 7.92–7.97 (d, 1H, benzene protons, *J =* 20 Hz), 8.21–8.24 (m, 1H, benzene protons), 8.52 (bs, 3H, NH_3_^+^), 10.37 (bs, 1H, CONH) ppm; ^13 ^C (100 MHz, DMSO-d_6_) *δ* 166.9, 140.6, 139.7, 137.8, 137.7, 133.4, 130.9, 130.2, 129.1 (2 C), 128.3 (2 C), 128.1, 127.9 (2 C), 127.7, 127.1, 121.3 (2 C), 34.7, 25.3, 19.6; MS (EI) *m/z*: 364.13 [M]^+^. Anal. (C_22_H_21_ClN_2_O) Calcd. (%): C, 72.42; H, 5.80; Cl, 9.72; N, 7.68. Found (%) C, 72.68; H, 5.94; Cl, 9.41; N, 7.35.

**N*-(4-(2-Aminocyclopropyl)phenyl)-4-(furan-2-yl)benzamide hydrochloride (2a).*** Chemical and physical data: m.p., >250 °C (acetonitrile/methanol); yield, 55%. ^1^H NMR (DMSO-d_6_, 400 MHz, *δ*; ppm) *δ* 1.19–1.21 (m, 1H, CHH cyclopropane), 1.37–1.39 (m, 1H, CHH cyclopropane), 2.32–2.33 (m, 1H, CHNH_2_ cyclopropane), 2.79–2.80 (m, 1H, PhCH cyclopropane), 6.68–6.70 (m, 1H, furan proton), 7.14–7.16 (m, 3H, furan and benzene protons), 7.72–7.74 (d, 2H, benzene protons, *J =* 8 Hz), 7.84–7.86 (m, 3H, furan and benzene protons), 8.02–8.04 (d, 2H, benzene protons, *J =* 8 Hz), 8.49 (bs, 3H, NH_3_^+^), 10.30 (bs, 1H, CONH) ppm; ^13 ^C (100 MHz, DMSO-d_6_) *δ* 168.2, 153.6, 142.3, 138.6, 138.4, 137.0, 134.4, 128.7 (2 C), 128.2 (2 C), 125.2 (2 C), 121.2 (2 C), 111.4, 107.7, 34.6, 25.3, 19.3; MS (EI) *m/z*: 354.11 [M]^+^. Anal. (C_20_H_19_ClN_2_O_2_) Calcd. (%): C, 67.70; H, 5.40; Cl, 9.99; N, 7.89. Found (%) C, 67.39; H, 5.27; Cl, 10.12; N, 7.94.

**N*-(4-(2-Aminocyclopropyl)phenyl)-3-(furan-2-yl)benzamide hydrochloride (2b).*** Chemical and physical data: m.p., 208–210 °C (acetonitrile/methanol); yield, 61%. ^1^H NMR (DMSO-d_6_, 400 MHz, *δ*; ppm) *δ* 1.20–1.24 (m, 1H, CHH cyclopropane), 1.35–1.40 (m, 1H, CHH cyclopropane), 2.28–2.33 (m, 1H, CHNH_2_ cyclopropane), 2.80–2.82 (m, 1H, PhCH cyclopropane), 6.65–6.66 (m, 1H, furan proton), 7.09–7.10 (d, 1H, furan proton, *J =* 4 Hz), 7.15–7.18 (d, 2H, benzene protons, *J =* 12 Hz), 7.57–7.60 (t, 1H, furan proton, *J =* 12 Hz), 7.69–7.74 (m, 2H, benzene protons), 7.74–7.79 (m, 2H, benzene protons), 7.94–7.96 (m, 1H, benzene proton), 8.25 (s, 1H, benzene protons), 8.39 (bs, 3H, NH_3_^+^), 10.37 (bs, 1H, CONH) ppm; ^13 ^C (100 MHz, DMSO-d_6_) *δ* 166.2, 154.0, 142.2, 138.6, 138.4, 133.1, 130.8, 130.7, 128.2, 128.2 (2 C), 128.0, 126.4, 121.2 (2 C), 111.5, 107.3, 34.6, 25.3, 19.3; MS (EI) *m/z*: 354.11 [M]^+^. Anal. (C_20_H_19_ClN_2_O_2_) Calcd. (%): C, 67.70; H, 5.40; Cl, 9.99; N, 7.89. Found (%) C, 67.52; H, 5.30; Cl, 10.07; N, 7.92.

**N*-(4-(2-Aminocyclopropyl)phenyl)-4-(furan-3-yl)benzamide hydrochloride (3a).*** Chemical and physical data: m.p., >250 °C (acetonitrile/methanol); yield, 75%. ^1^H NMR (DMSO-d_6_, 400 MHz, *δ*; ppm) *δ* 1.19–1.21 (m, 1H, CHH cyclopropane), 1.38–1.39 (m, 1H, CHH cyclopropane), 2.29–2.31 (m, 1H, CHNH_2_ cyclopropane), 2.80 (bs, 1H, PhCH cyclopropane), 7.09 (s, 1H, furan proton), 7.14–7.16 (d, 2H, benzene proton, *J =* 8 Hz), 7.72–7.73 (d, 2H, benzene proton, *J =* 4 Hz), 7.79–7.81 (m, 3H, furan and benzene proton), 7.96–7.98 (d, 2H, benzene proton, *J =* 8 Hz), 8.36 (s, 1H, benzene proton), 8.47 (bs, 3H, NH_3_^+^), 10.26 (bs, 1H, CONH) ppm; ^13 ^C (100 MHz, DMSO-d_6_) *δ* 168.2, 143.0, 140.8, 138.6, 138.4, 137.8, 136.6, 130.1 (2 C), 128.2 (2 C), 127.2, 126.6 (2 C), 121.2 (2 C), 108.8, 34.6, 25.3, 19.3; MS (EI) *m/z*: 354.11 [M]^+^. Anal. (C_20_H_19_ClN_2_O_2_) Calcd. (%): C, 67.70; H, 5.40; Cl, 9.99; N, 7.89. Found (%) C, 67.98; H, 5.41; Cl, 9.79; N, 7.54.

**N*-(4-(2-Aminocyclopropyl)phenyl)-3-(furan-3-yl)benzamide hydrochloride (3b).*** Chemical and physical data: m.p., 235–238 °C (acetonitrile/methanol); yield, 71%. ^1^H NMR (DMSO-d_6_, 400 MHz, *δ*; ppm) *δ* 1.20–1.21 (m, 1H, CHH cyclopropane), 1.38–1.39 (m, 1H, CHH cyclopropane), 2.29–2.31 (m, 1H, CHNH_2_ cyclopropane), 2.79–2.81 (m, 1H, PhCH cyclopropane), 7.08 (s, 1H, furan proton), 7.15–7.17 (d, 2H, benzene protons, *J =* 8 Hz), 7.53–7.55 (t, 1H, furan proton, *J =* 8 Hz), 7.71–7.73 (d, 2H, benzene proton, *J =* 8 Hz), 7.80–7.81 (m, 3H, furan and benzene proton), 8.17 (s, 1H, benzene proton), 8.32 (s, 1H, benzene proton), 8.45 (bs, 3H, NH_3_^+^), 10.33 (bs, 1H, CONH) ppm; ^13 ^C (100 MHz, DMSO-d_6_) *δ* 166.4, 141.9, 140.7, 138.6, 138.4, 133.9, 133.9, 130.3, 129.9, 128.5, 128.2 (2 C), 127.4, 126.8, 121.2 (2 C), 109.5, 34.6, 25.3, 19.3; MS (EI) *m/z*: 354.11 [M]^+^. Anal. (C_20_H_19_ClN_2_O_2_) Calcd. (%): C, 67.70; H, 5.40; Cl, 9.99; N, 7.89. Found (%) C, 67.65; H, 5.31; Cl, 10.06; N, 7.99.

**N*-(4-(2-Aminocyclopropyl)phenyl)-4-(thiophen-2-yl)benzamide hydrochloride (4a).*** Chemical and physical data: m.p., >250 °C (methanol); yield, 82%. ^1^H NMR (DMSO-d_6_, 400 MHz, *δ*; ppm) *δ* 1.17–1.24 (m, 1H, CHH cyclopropane), 1.36–1.41 (m, 1H, CHH cyclopropane), 2.30–2.35 (m, 1H, CHNH_2_ cyclopropane), 2.77–2.81 (m, 1H, PhCH cyclopropane), 7.14–7.17 (m, 2H, thiophen protons), 7.19–7.21 (m, 1H, thiophen proton), 7.65–7.67 (m, 1H, benzene protons), 7.68–7.69 (m, 1H, benzene protons), 7.72–7.74 (d, 2H, benzene protons, *J =* 8 Hz), 7.81–7.83 (d, 2H, benzene protons, *J =* 8 Hz), 8.01–8.03 (d, 2H, benzene protons, *J =* 8 Hz), 8.42 (bs, 3H, NH_3_^+^), 10.23 (bs, 1H, CONH) ppm; ^13 ^C (100 MHz, DMSO-d_6_) *δ* 168.2, 143.0, 139.2, 138.6, 138.4, 136.9, 128.6 (2 C), 128.2 (2 C), 127.9, 126.9, 126.8 (2 C), 124.7, 121.2 (2 C), 34.6, 25.3, 19.3; MS (EI) *m/z*: 370.09 [M]^+^. Anal. (C_20_H_19_ClN_2_OS) Calcd. (%): C, 64.77; H, 5.16; Cl, 9.56; N, 7.55; S, 8.64. Found (%) C, 64.43; H, 5.09; Cl, 9.68; N, 7.84; S, 8.49.

**N*-(4-(2-Aminocyclopropyl)phenyl)-3-(thiophen-2-yl)benzamide hydrochloride (4b).*** Chemical and physical data: m.p., 245–248 °C (methanol); yield, 67%. ^1^H NMR (DMSO-d_6_, 400 MHz, *δ*; ppm) *δ* 1.17–1.23 (m, 1H, CHH cyclopropane), 1.35–1.40 (m, 1H, CHH cyclopropane), 2.29–2.34 (m, 1H, CHNH_2_ cyclopropane), 2.79–2.81 (m, 1H, PhCH cyclopropane), 7.16–7.21 (m, 3H, thiophene and benzene protons), 7.56–7.60 (t, 1H, thiophene protons, *J =* 16 Hz), 7.62–7.66 (m, 2H, thiophene and benzene protons), 7.72–7.74 (d, 2H, benzene protons, *J =* 8 Hz), 7.86–7.89 (d, 2H, benzene protons, *J =* 12 Hz), 8.18–8.20 (m, 1H, benzene protons), 8.37 (bs, 3H, NH_3_^+^), 10.37 (bs, 1H, CONH) ppm; ^13 ^C (100 MHz, DMSO-d_6_) *δ* 165.9, 141.7, 138.6, 138.4, 135.9, 132.8, 130.3, 128.5, 128.3, 128.2 (2 C), 128.1, 127.8, 126.5, 124.4, 121.2 (2 C), 34.6, 25.3, 19.3; MS (EI) *m/z*: 370.109 [M]^+^. Anal. (C_20_H_19_ClN_2_OS) Calcd. (%): C, 64.77; H, 5.16; Cl, 9.56; N, 7.55; S, 8.64. Found (%) C, 64.94; H, 5.28; Cl, 9.45; N, 7.42; S, 8.77.

**N*-(4-(2-Aminocyclopropyl)phenyl)-4-(thiophen-3-yl)benzamide hydrochloride (5a).*** Chemical and physical data: m.p., 222–225 °C (methanol); yield, 81%. ^1^H NMR (DMSO-d_6_, 400 MHz, *δ*; ppm) *δ* 1.18–1.20 (m, 1H, CHH cyclopropane), 1.21–1.23 (m, 1H, CHH cyclopropane), 2.28–2.33 (m, 1H, CHNH_2_ cyclopropane), 2.80 (bs, 1H, PhCH cyclopropane), 7.15–7.19 (d, 2H, benzene protons, *J =* 16 Hz), 7.67–7.75 (m, 4H, thiophene and benzene protons), 7.89–7.94 (d, 2H, benzene protons, *J =* 20 Hz), 8.05–8.09 (d, 2H, benzene protons, *J =* 16 Hz), 8.14–8.16 (m, 1H, thiophene proton), 8.40 (bs, 3H, NH_3_^+^), 10.28 (bs, 1H, CONH) ppm; ^13 ^C (100 MHz, DMSO-d_6_) *δ* 168.5, 155.4, 141.7, 140.5, 139.1, 137.9, 136.8, 129.7 (2 C), 127.7, 127.3, 127.2, 127.1, 122.4, 121.2, 79.7, 33.8, 28.3, 26.3, 18.1; MS (EI) *m/z*: 370.09 [M]^+^. Anal. (C_20_H_19_ClN_2_OS) Calcd. (%): C, 64.77; H, 5.16; Cl, 9.56; N, 7.55; S, 8.64. Found (%) C, 65.07; H, 5.24; Cl, 9.39; N, 7.22; S, 8.78.

**N*-(4-(2-Aminocyclopropyl)phenyl)-3-(thiophen-3-yl)benzamide hydrochloride (5b).*** Chemical and physical data: m.p., >250 °C (methanol); yield, 85%. ^1^H NMR (DMSO-d_6_, 400 MHz, *δ*; ppm) *δ* 1.20–1.21 (m, 1H, CHH cyclopropane), 1.35–1.38 (m, 1H, CHH cyclopropane), 2.29–2.32 (m, 1H, CHNH_2_ cyclopropane), 2.81 (bs, 1H, PhCH cyclopropane), 7.17–7.18 (d, 2H, thiophene proton, *J =* 4 Hz), 7.49–7.51 (t, 1H, thiophene proton, *J =* 8 Hz), 7.67–7.69 (m, 1H, benzene protons), 7.70–7.73 (m, 3H, thiophene and benzene protons), 7.84– 7.86 (d, 1H, benzene proton, *J =* 8 Hz), 7.90–7.92 (d, 1H, benzene proton, *J =* 8 Hz), 8.02 (bs, 1H, benzene proton), 8.26 (s, 1H, benzene proton), 8.40 (bs, 3H, NH_3_^+^), 10.34 (bs, 1H, CONH) ppm; ^13 ^C (100 MHz, DMSO-d_6_) *δ* 166.9, 140.8, 138.6, 137.8, 137.7, 133.6, 130.3, 130.2, 128.4, 128.3 (2 C), 127.4, 127.1, 127.1, 122.6, 121.3 (2 C), 34.7, 25.3, 19.6; MS (EI) *m/z*: 370.09 [M]^+^. Anal. (C_20_H_19_ClN_2_OS) Calcd. (%): C, 64.77; H, 5.16; Cl, 9.56; N, 7.55; S, 8.64. Found (%) C, 64.92; H, 5.23; Cl, 9.47; N, 7.69; S, 8.47.

**N*-(4-(2-Aminocyclopropyl)phenyl)-4-(pyridin-4-yl)benzamide hydrochloride (6a).*** Chemical and physical data: m.p., >250 °C (methanol); yield, 48%. ^1^H NMR (DMSO-d_6_, 400 MHz, *δ*; ppm) *δ* 1.17–1.22 (m, 1H, CHH cyclopropane), 1.39–1.44 (m, 1H, CHH cyclopropane), 2.33–2.39 (m, 1H, CHNH_2_ cyclopropane), 2.77–2.80 (m, 1H, PhCH cyclopropane), 7.16–7.18 (d, 2H, benzene protons, *J =* 8 Hz), 7.75–7.77 (d, 2H, benzene protons, *J =* 8 Hz), 8.14–8.20 (m, 4H, benzene protons), 8.30–8.32 (d, 2H, pyridine protons, *J =* 8 Hz), 8.58 (bs, 3H, NH_3_^+^), 8.93–8.95 (d, 2H, pyridine protons, *J =* 8 Hz), 10.49 (bs, 1H, CONH) ppm; ^13 ^C (100 MHz, DMSO-d_6_) *δ* 168.5, 148.2 (2 C), 147.5, 141.8, 137.9, 137.7, 136.8, 129.6 (2 C), 128.4 (2 C), 128.3 (2 C), 121.6 (2 C), 121.3 (2 C), 34.7, 25.3, 19.6; MS (EI) *m/z*: 365.13 [M]^+^. Anal. (C_21_H_20_ClN_3_O) Calcd. (%): C, 68.94; H, 5.51; Cl, 9.69; N, 11.49. Found (%) C, 69.17; H, 5.64; Cl, 9.49; N, 11.38.

**N*-(4-(2-Aminocyclopropyl)phenyl)-3-(pyridin-4-yl)benzamide hydrochloride (6b).*** Chemical and physical data: m.p., >250 °C (methanol); yield, 63%. 1H NMR (DMSO-d_6_, 400 MHz, *δ*; ppm) *δ* 1.18–1.23 (m, 1H, CHH cyclopropane), 1.37–1.42 (m, 1H, CHH cyclopropane), 2.32–2.35 (m, 1H, CHNH_2_ cyclopropane), 2.79–2.82 (m, 1H, PhCH cyclopropane), 7.17–7.19 (d, 2H, benzene protons, *J =* 8 Hz), 7.76–7.79 (t, 3H, benzene protons, *J =* 12 Hz), 8.15–8.21 (m, 2H, pyridine protons), 8.39–8.40 (d, 2H, benzene protons, *J =* 4 Hz), 8.50 (bs, 3H, NH_3_^+^), 8.55 (s, 1H, benzene protons), 8.95–8.97 (d, 2H, pyridine protons, *J =* 8 Hz), 10.58 (bs, 1H, CONH) ppm; ^13 ^C (100 MHz, DMSO-d_6_) *δ* 166.9, 150.2 (2 C), 146.2, 139.3, 137.8, 137.7, 133.4, 131.5, 130.2, 128.3, 128.1 (2 C), 128.0, 121.8 (2 C), 121.3 (2 C), 34.7, 25.3, 19.6; MS (EI) *m/z*: 365.13 [M]^+^. Anal. (C_21_H_20_ClN_3_O) Calcd. (%): C, 68.94; H, 5.51; Cl, 9.69; N, 11.49. Found (%) C, 69.22; H, 5.68; Cl, 9.54; N, 11.31.

### 2.4. Lsd1-CoREST binding and inhibition assays

Thermal stability, activity on histone H3K4me peptide, and inhibition of human LSD1-CoREST were measured using established protocols^17^^,^^28^.

The complex of human recombinant LSD1/CoREST protein was produced in *E. coli* as separate proteins and co-purified following previously reported procedures^13^^,^^29^. The experiments were performed in 96 well half area white plates (cat. 3693, Corning, Corning, NY) using a di-methylated H3K4 peptide containing 21 amino acids (custom synthesis done by Thermo Scientific) as substrate in 100 μL volume of 50 mM HEPES, pH 7.5. The peptide purity was >95% as checked by analytical high-pressure liquid chromatography and mass spectrometry. The demethylase activity was estimated under aerobic conditions at room temperature by measuring the release of H_2_O_2_ produced during the catalytic process by the Amplex UltraRed detection system coupled with horseradish peroxidase (HRP). Briefly, 300 nM of LSD1/CoREST complex was incubated at room temperature for 10 min in the absence and/or the presence of various concentrations of the inhibitors, 50 mM Amplex UltraRed (Life Technologies) and 0.023 mM HRP (Sigma-Aldrich, Schnellendorf, Germany) in 50 mM HEPES pH 7.5 and 0.05 mg/mL BSA. The inhibitors were tested twice in duplicates at each concentration. Tranylcypromine (Sigma) was used as a control. After preincubation of the enzyme with the inhibitor, the reaction was initiated by adding 10 μМ of the dimethylated H3K4 peptide. The conversion of the Amplex UltraRed reagent to resurfin was monitored by fluorescence (excitation at 510 nm, emission at 595 nm). Arbitrary units were used to measure the level of H_2_O_2_ produced in the absence and/or in the presence of inhibition. The maximum demethylase activity of LSD1/CoREST was obtained in the absence of inhibitors and corrected for background fluorescence in the absence of the substrate. The IC_50_ values were calculated using GraphPad Prism version 4.0 (GraphPad Software, San Diego, CA).

### 2.5. Anti-MAO assays

The tested compounds were dissolved in DMSO (Sigma-Aldrich, Schnellendorf, Germany) to prepare 10 mM stock solutions which were kept for storage at −20 °C. The percentage of DMSO used in the experiments was never higher than 1%. Clorgyline and selegiline, used as reference inhibitors, have been acquired from Sigma-Aldrich, Schnellendorf, Germany. Human recombinant MAO isoforms, used in the experiments, was purchased from Sigma-Aldrich. Resorufin sodium salt, *p*-tyramine hydrochloride, sodium phosphate buffer, horseradish peroxidase, and Amplex Red reagent have been supplied in the assay kit of Amplex Red MAO Molecular Probes (Molecular Probes Inc., Eugene, OR). Determination of MAO isoforms enzymatic activity: Briefly, 0.1 ml of sodium phosphate buffer (0.05 M, pH 7.4) containing different concentrations of the test drugs (new compounds or reference inhibitors) in various concentrations and adequate amounts of recombinant hMAO-A or hMAO-B required and adjusted to obtain in our experimental conditions the same reaction velocity, that is, to oxidise (in the control group) the same concentration of substrate: 165 pmol of *p*-tyramine/min (hMAO-A: 1.1 μg protein; specific activity: 150 nmol of *p*-tyramine oxidised to *p*-hydroxyphenylacetaldehyde/min/mg protein; hMAO-B: 7.5 μg protein; specific activity: 22 nmol of *p*-tyramine transformed/min/mg protein) were incubated for 15 min at 37 °C in a flat-black-bottom 96-well microtest plate, placed in the dark fluorimeter chamber. After this incubation period, the reaction was started by adding (final concentrations) 200 μM Amplex Red reagent, 1 U/mL horseradish peroxidase, and 1 mM *p*-tyramine. The production of H_2_O_2_ and, consequently, of resorufin was quantified at 37 °C in a multidetection microplate fluorescence reader (FLX800, Bio-Tek Instruments Inc., Winooski, VT) based on the fluorescence generated (excitation, 545 nm, emission, 590 nm) over a 15 min period, in which the fluorescence increased linearly. Control experiments were carried out simultaneously by replacing the tested drugs (new compounds and reference inhibitors) with appropriate dilutions of the vehicles. In addition, the possible capacity of the above test drugs to modify the fluorescence generated in the reaction mixture due to non-enzymatic inhibition (e.g. for directly reacting with Amplex Red reagent) was determined by adding these drugs to solutions containing only the Amplex Red reagent in a sodium phosphate buffer. To determine the kinetic parameters of hMAO-A and hMAO-B (K_m_ and V_max_), the corresponding enzymatic activity of both isoforms was evaluated (under the experimental conditions described above) in the presence of a wide range of *p*-tyramine concentrations. The specific fluorescence emission (used to obtain the final results) was calculated after subtraction of the background activity, which was determined from wells containing all components except the hMAO isoforms, which were replaced by a sodium phosphate buffer solution. In our experimental conditions, this background activity was practically negligible. MAO activity of the test compounds and reference inhibitors is expressed as IC_50_, i.e. the concentration of each drug required to result in a 50% decrease in respect to the control value activity of the relative MAO isoforms. The corresponding IC_50_ values were calculated by using the Origin 5.0 software (Microcal Software Inc., Northampton, MA).

### 2.6. Cellular assays

#### 2.6.1. Chemicals

The tested compounds have been dissolved in DMSO at 50 μM as stock concentration. All compounds have been tested at 10 μM and 50 μM as final concentrations for WB analysis. All compounds have been used at 0.1 μM, 5 μM, 10 μM, 25 μM, 50 μM as final concentration for proliferation assays. ORY-1001 (Selleck Catalog No. S7795) was used as reference compound at the concentration of 25 μM.

#### 2.6.2. Cell cultures

Cell culture LNCaP and U937 were purchased from ATCC (Milan, Italy). LNCaP were grown in Roswell Park Memorial Institute culture medium (RPMI; EuroClone, Milan, Italy), supplemented with 10% heat-inactivated foetal bovine serum (FBS; Sigma-Aldrich, Schnellendorf, Germany), antimicrobials (100 U/mL penicillin, 100 µg/mL streptomycin, 250 ng/mL amphotericin-B), 2 mM L-glutamine (EuroClone, Milan, Italy), and 1% essential amino acids solution (MEM; EuroClone, Milan, Italy). U937 were cultured in Dulbecco’s Modified Eagle Medium (DMEM; EuroClone, Milan, Italy), supplemented with 10% heat-inactivated foetal bovine serum (FBS; Sigma-Aldrich, Schnellendorf, Germany), antimicrobials (100 U/mL penicillin, 100 µg/mL streptomycin, 250 ng/mL amphotericin-B), 2 mM L-glutamine (EuroClone). Cells have grown in standard incubator conditions at 37 °C and 5% CO_2_.

#### 2.6.3. Histone extraction, Western blot analysis and protein extraction

After stimulation for 48 h with the compounds at 10 μM and 50 μM, ORY-1001 (Selleck Catalog No. S7795), commercially available LSD1 inhibitor, was used as positive controls. ORY-1001 was used at the final concentration of 25 μM. Cells were collected and washed 2 times with PBS then processed for histone extraction. Pellets were resuspended in triton extraction buffer [TEB; PBS containing 0.5% Triton X 100 (v/v), 2 mmol/L PMSF, 0.02% (w/v) NaN_3_], and the lysis was performed for 10 min at 4 °C. The samples were centrifuged at 2000× *g* for 10 min at 4 °C and pellets were washed in TEB (half volume). Samples were then resuspended in 0.2 N HCl, and acid histone extraction was carried out overnight at 4 °C. The supernatants were recovered, and protein concentration was quantified by Bradford assay (Bio-Rad). For each sample, 4 μg of proteins were loaded on 15% polyacrylamide gels. The nitrocellulose filters were stained with Ponceau red (Sigma-Aldrich, Schnellendorf, Germany) as an additional control for equal loading. H3K4me2, H3K9me2 (Diagenode, Ougrée, Belgium; pAB-035–050, pAb-060–050), and H4 (Cell Signalling #2592) were used according to the manufacturer’s instructions.

#### 2.6.4. Statistical analysis and software tool analysis

Raw data were acquired by using ImageJ software and a semi-quantitative analysis was performed. Relative intensities were normalised on the control and reported above images, in Figure 5.

#### 2.6.5. Proliferative assay

Cell viability was determined on LNCaP and U937 cell lines using Thiazolyl Blue Tetrazolium Bromide [3-(4,5-dimethylthiazol-2-yl)-2,5-diphenyltetrazolium bromide] (MTT; Sigma-Aldrich, Schnellendorf, Germany) assay, following manufacturer’s instructions. A total of 5 × 10^3^ cells/well were plated in a 96-well plate and treated, in duplicates, and repeated for three times, with the tested compounds at different concentrations 0.1 μM, 5 μM, 10 μM, 25 μM, 50 μM as final concentrations for 48 h, and 72 h. Tranylcypromine (TCP) and ORY-1001 were used as positive controls (data not shown). TCP was purchased from Sigma-Aldrich (St Louis, MO) and used at a final concentration of 100 μM and ORY-1001 at 25 μM. Absorbance was read at a wavelength of 570 nm with a TECAN M − 200 reader (Tecan, Männedorf, Switzerland).

## 3. Results and discussion

### 3.1. Chemistry

**Scheme 1.**
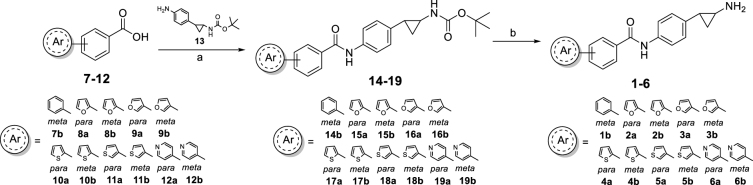
Synthesis of the *N*-(4-(2-aminocyclopropyl)phenyl)-(hetero)aryl-4- and 3-carboxamides **1 b**-**6**. Reagents and conditions: (a) PyBop, Et_3_N, dry DMF, N_2_ atmosphere, overnight, rt. (b) 4 N HCl, dry dioxane/THF, overnight, rt.

The *tert*-butyl carbamates **14**–**19**, key intermediates for the synthesis of the final compounds **1**–**6**, were prepared by coupling the *tert*-butyl (2-(4-aminophenyl)cyclopropyl)carbamate) **13**^17^ with the appropriate 4- or 3-substituted benzoic acids **7**–**12**, all commercially available, in the presence of benzotriazol-1-yloxytripyrrolidinophosphonium hexafluorophosphate (PyBOP) and triethylamine, using dry *N*,*N*-dimethylformamide as the solvent. The subsequent cleavage of the Boc protection from the carbamates **14**–**19** with 4 N HCl in dioxane and dry THF afforded the *N*-(4-(2-aminocyclopropyl)phenyl)-(hetero)aryl-4- and 3-carboxamides **1**–**6** as hydrochloride salts (Scheme 1).

### 3.2. Lsd1 and MAO enzymes evaluation assays

**Figure 3.**
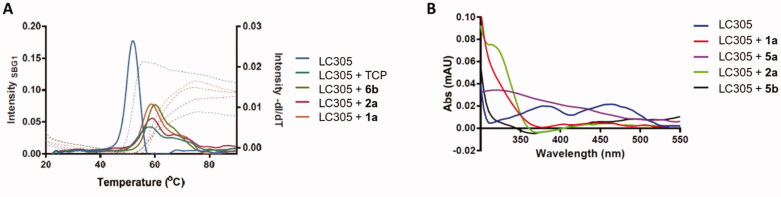
Representative samples of thermal shifts by ThermoFAD assay (A) and FAD spectral bleaching (B) for selected compounds. LC305 is the code for the LSD1-CoREST complex.

First, the new TCP-based compounds **1b**-**6** were tested to detect their binding and inhibition of LSD1. ThermoFAD assay^28^ was used to detect the effects of **1b**-**6** on the thermal stability of the LSD1-CoREST complex, and the largest shifts in the Tm of the enzyme usually indicate the strongest LSD1 binding abilities (Table 1). Representative curves relative to ThermoFAD assay and FAD spectral bleaching using LSD1-CoREST (LC305) with selected compounds have been depicted in Figure 3(A,B), respectively.

**Table 1.** ThermoFAD assay and inhibition values (IC_50_ values) of **1 b**-**6** vs LSD1-CoREST complex.

Lab code	compd	LSD1-CoREST	
		Thermal shift, °C	IC_50_, μM^a^
MC2652	**1a**	+ 6.5	0.190 ± 0.030
MC3993	**1b**	+ 8.0	0.149 ± 0.043
MC4014	**2a**	+ 7.0	2.001 ± 0.528
MC4032	**2b**	+ 8.0	0.110 ± 0.044
MC4022	**3a**	+ 6.5	2.343 ± 0.225
MC4024	**3b**	+ 8.0	0.221 ± 0.023
MC3995	**4a**	+ 6.5	1.077 ± 0.359
MC3994	**4b**	+ 8.0	0.015 ± 0.003
MC4030	**5a**	+ 5.0	0.341 ± 0.106
MC4025	**5b**	+ 7.5	0.005 ± 0.0008
MC4031	**6a**	+ 7.0	13.94 ± 3.06
MC4036	**6b**	+ 8.0	0.572 ± 0.157
	TCP	+ 6.5	40.82 ± 3.11

TCP and **1a** were used as reference compounds.

^a^Each IC_50_ value is the mean ± SD from two experiments in duplicate.

**Figure 4.**
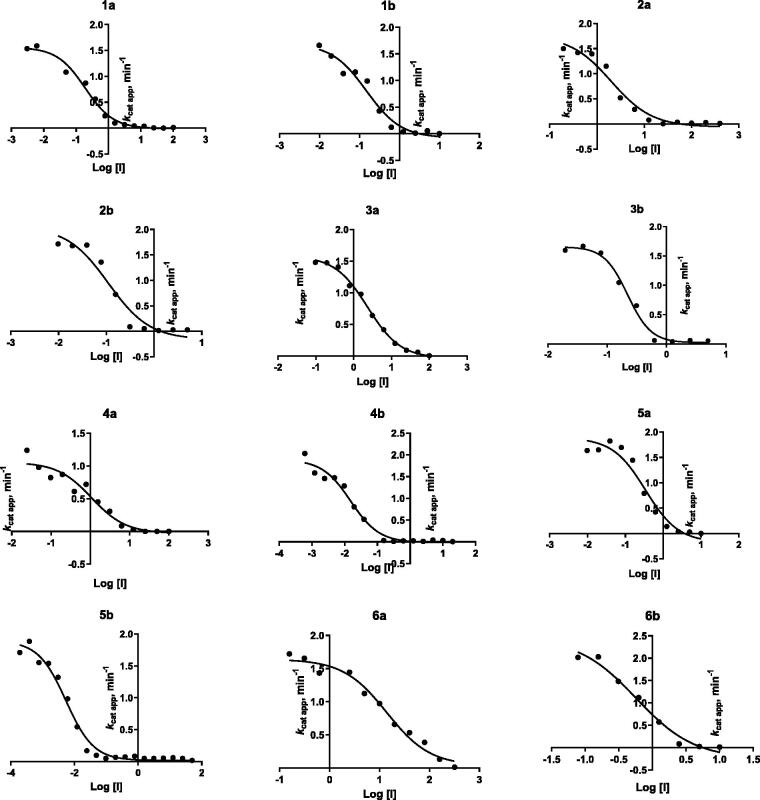
Inhibition curves of **1 b**-**6** against LSD1-CoREST. Compound **1a** has been reported for comparison purpose.

The inhibition of LSD1 was assessed by protein expression in *Escherichia coli* and co-purification of LSD1*Δ*124-CoREST1*Δ*305 (LSD1-CoREST) and using H3K4me1 (residues 1–21) peptide as the substrate, as previously described^17^. The IC_50_ values of **1b**-**6** against the LSD1-CoREST complex are reported in Table 1, and the relative curves are depicted in Figure 4. TCP and **1a** were used as reference compounds.

The shift of the 4-phenyl ring of the benzoylamino moiety of **1a** from the *para* to *meta* position led to slightly increased potency (1.3-fold) against LSD1 (compare **1b** with **1a**). This trend of inhibition potency is confirmed for all the following derivatives, in which the phenyl ring has been replaced by a 2-furyl (**2a,b**), 3-furyl (**3a,b**), 2-thienyl (**4a,b**), 3-thienyl (**5a,b**), and 4-pyridyl (**6a,b**) group. In general, the *meta* regioisomers **2b**, **3b**, **4b**, **5b** and **6b** displayed 10.6- to 71.8-fold higher potency against LSD1 than the *para* counterparts. When compared to the phenyl-containing **1a** and **1b**, the 2-furyl (**2a,b**) compounds decreased the inhibitory potency when placed in *para* (**2a**), and gave little improvement when inserted in *meta* (**2b**). Differently, the replacement with a 3-furyl (**3a,b**) ring was detrimental for the anti-LSD1 activity regardless of the *para* or *meta* position. The introduction of the 2- or 3-thienyl (**4a,b** and **5a,b**) ring led to a severe (1.8- to 5.7-fold) drop of potency when the heterocycle was placed in *para* (compare **4a** and **5a** with **1a**), whereas the *meta* corresponding analogs **4b** and **5b** showed 10- to 30-fold improved potency respect to **1b** in the anti-LSD1 assay. At last, the 4-pyridyl (**6a,b**) ring abated the inhibitory potency from 73 (**6a**) to 4 (**6b**) times when compared with **1a** and **1b**, respectively.

Since **1b-6** contain in their structure the TCP fragment, they were tested against MAOs to assess their eventual LSD1 selectivity. The IC_50_ values of **1b**-**6** against MAO-A and MAO-B have been summarised in Table 2. Clorgyline and *R*-(-)-deprenyl were used as reference MAO-A and -B selective inhibitors, respectively.

**Table 2.** Anti-MAO activities of **1 b**-**6**.^a^

Lab code	compd	IC_50_, μM		MAO Selectivity Index^b^
		MAO-A	MAO-B	
MC3993	**1b**	0.028 ± 0.002	0.327 ± 0.028	11.7
MC4014	**2a**	0.043 ± 0.004	0.073 ± 0.006	1.7
MC4032	**2b**	0.093 ± 0.008	0.888 ± 0.077	9.5
MC4022	**3a**	0.160 ± 0.014	0.093 ± 0.008	0.6
MC4024	**3b**	1.120 ± 0.097	0.519 ± 0.036	0.5
MC3995	**4a**	0.025 ± 0.002	0.032 ± 0.003	1.3
MC3994	**4b**	0.024 ± 0.002	0.366 ± 0.032	15.3
MC4030	**5a**	0.042 ± 0.004	0.067 ± 0.006	1.6
MC4025	**5b**	0.028 ± 0.002	0.820 ± 0.071	29.3
MC4031	**6a**	0.069 ± 0.006	0.358 ± 0.031	5.2
MC4036	**6b**	0.159 ± 0.014	10.42 ± 0.30	66
	clorgyline	0.005 ± 0.0009	63.41 ± 1.20	12,682
	*R*-(-)-deprenyl	68.73 ± 4.21	0.017 ± 0.002	0.0002

^a^Each IC_50_ value is the mean ± SD from three experiments (*n* = 3).

^b^IC_50_^MAO-B^/IC_50_^MAO-A^.

The tested compounds displayed IC_50_ values against MAO-A in the range 0.024–1.120 μM, and against MAO-B they were often less potent, with a range of inhibition between 0.032 and 10.42 μM. Among the *para* and *meta* (**a** and **b**) substituted analogs, the first generally were more potent than the latter against the two MAO isoforms, reversing the behaviour observed with LSD1. Exceptions to this rule are the thienyl derivatives (**4a,b** and **5a,b**) versus MAO-A: indeed, against this MAO isoform, the 2-thienyl derivatives **4a** and **4b** showed the same inhibitory activity, and the 3-thienyl *meta* derivative **5b** was 1.5-fold more potent than the corresponding *para* analog **5a**.

Comparing the activities of **1b**-**6** against LSD1 and MAOs, all the *meta*-substituted derivatives showed from 2.2- to 164-fold selectivity for LSD1 vs MAO-B, and some of them (**3b**-**5b**) also vs MAO-A (Table 3). The *meta* 2- and 3-thienyl compounds **4b** and **5b** emerged as the most potent against LSD1 (IC_50_ values = 0.015 (**4b**) and 0.005 (**5b**) μM) and the most LSD1-selective respect to MAO-B (selectivity indexes = 24.4 (**4b**) and 164 (**5b**)).

**Table 3.** LSD1 selectivity over MAOs.

Lab code	compd	LSD1 selectivity vs MAO-A^a^	LSD1 selectivity vs MAO-B^b^
MC3993	**1b**	0.2	2.2
MC4032	**2b**	0.8	8.1
MC4024	**3b**	5.1	2.3
MC3994	**4b**	1.6	24.4
MC4025	**5b**	5.6	164.0
MC4036	**6b**	0.3	18.2

^a^IC_50_^MAO-A^/IC_50_^LSD1^.

^b^IC_50_^MAO-B^/IC_50_^LSD1^.

### 3.3. Effects of 1a,b, 2b, 3b, 4b, and 5a,b in U937 AML and prostate cancer LNCaP cells

Selected compounds among the most potent against LSD1 at enzyme level (**1a,b**, **2b**, **3b**, **4b**, and **5a,b**, with IC_50_ values in the range 0.005–0.341 μM) were tested at 0.1, 5, 10, 25, and 50 μM for 48 and 72 h in U937 AML and prostate cancer LNCaP cells to determine their effects on cell viability. Tables 4 and 5 show the dose-dependent decrease of cell viability observed after treatment in U937 and LNCaP cells, respectively.

**Table 4.** Effects of **1a,b**, **2 b**, **3 b**, **4 b**, and **5a,b** on cell viability in U937 cells after 48 and 72 h of treatment.

**48 h**			
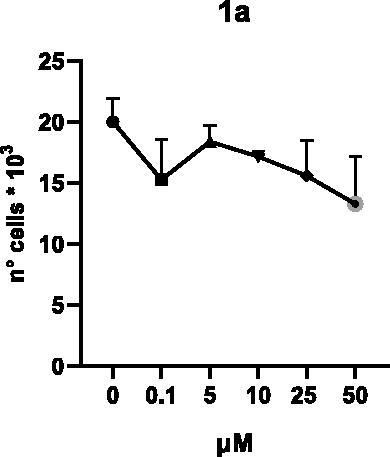	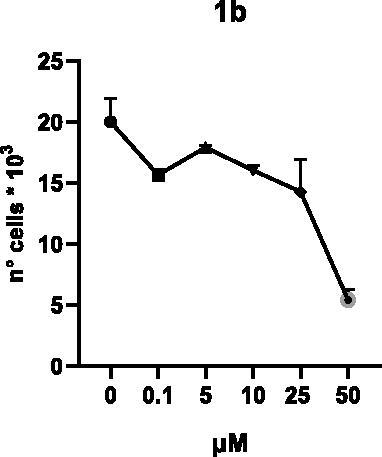	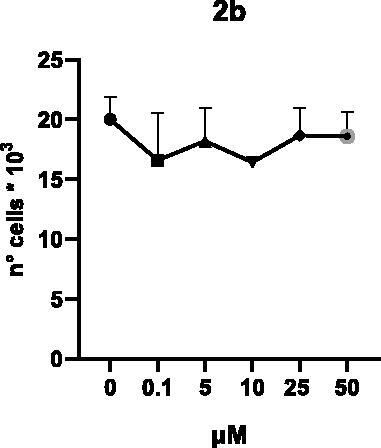	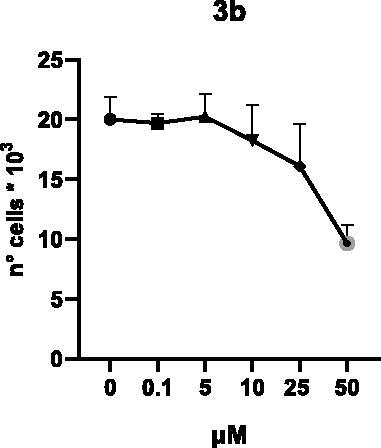
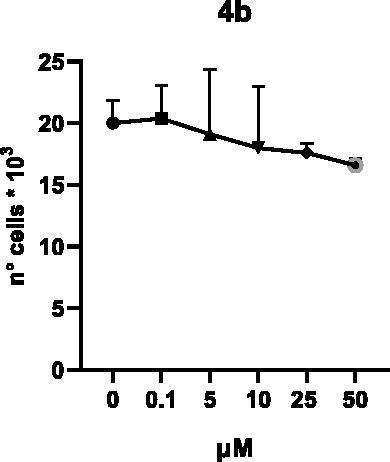	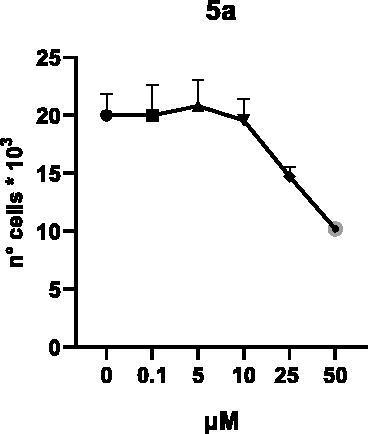	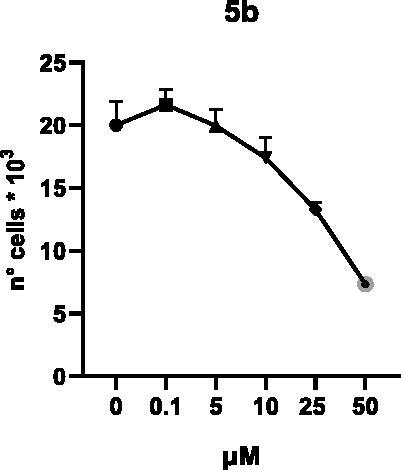	
**72 h**			
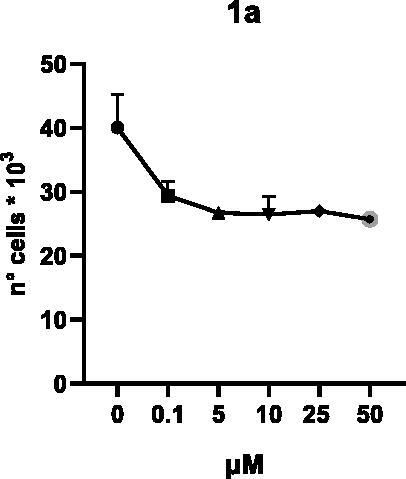	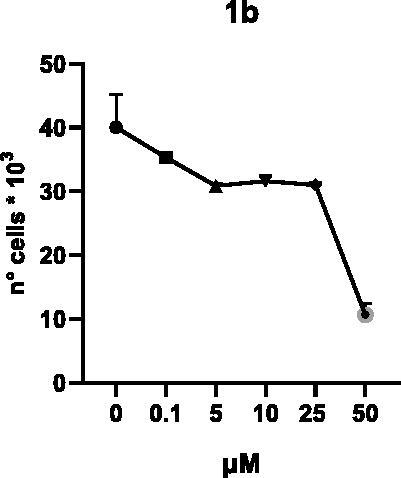	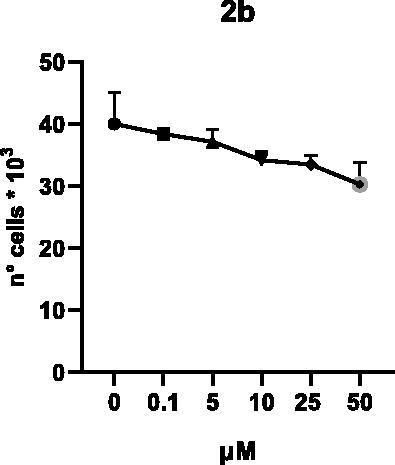	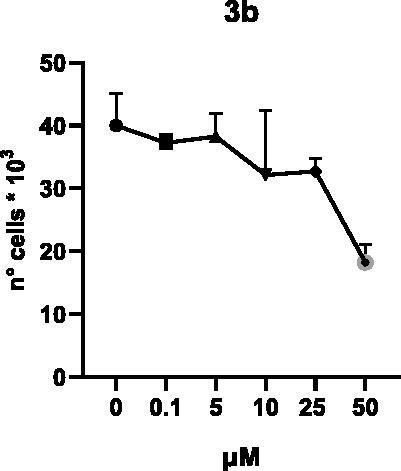
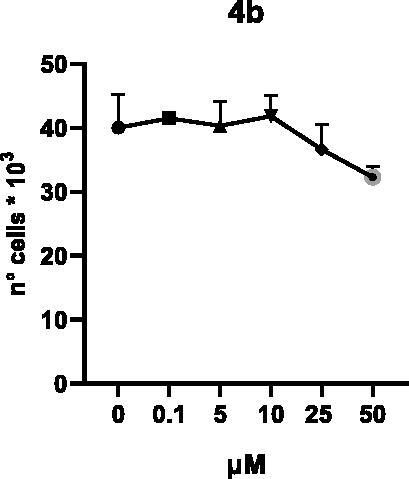	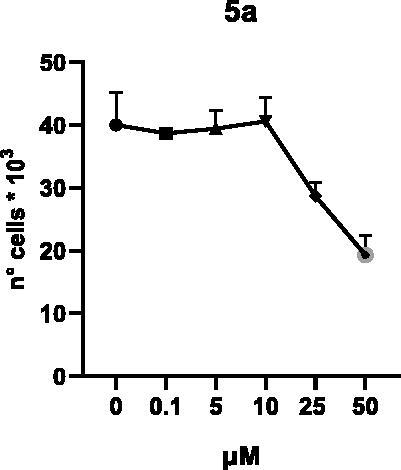	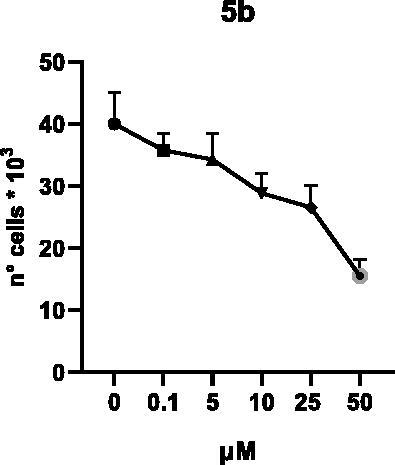	

**Table 5.** Effects of **1a,b**, **2 b**, **3 b**, **4 b**, and **5a,b** on cell viability in LNCaP cells after 48 and 72 h of treatment.

**48 h**			
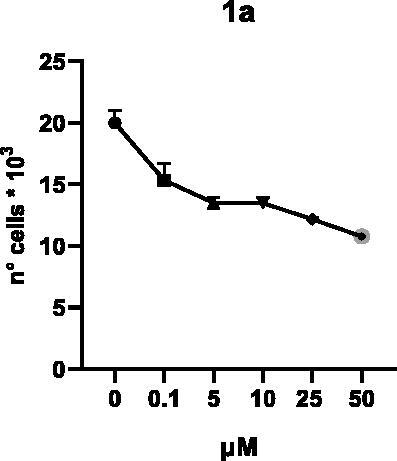	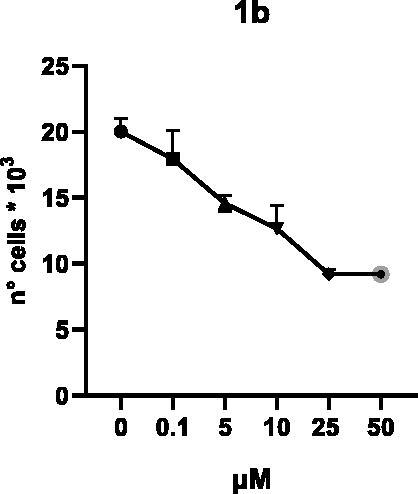	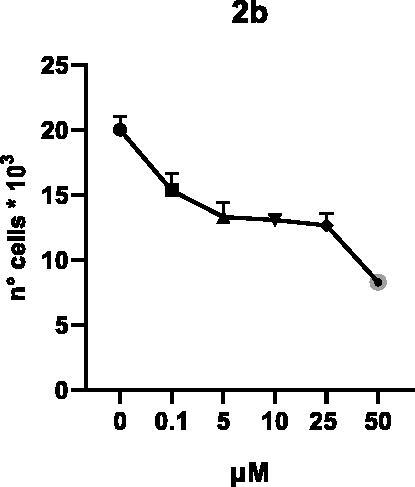	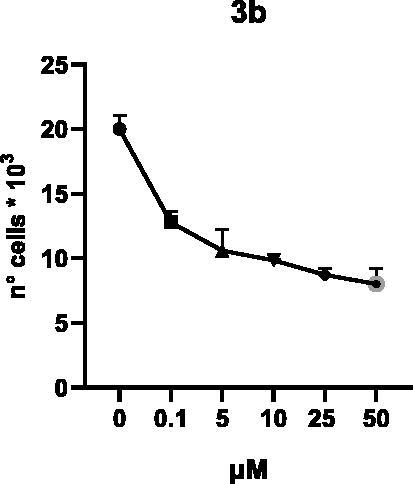
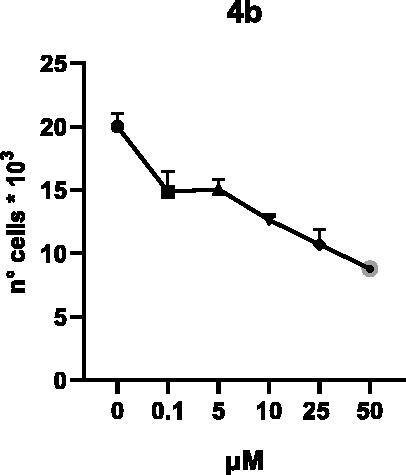	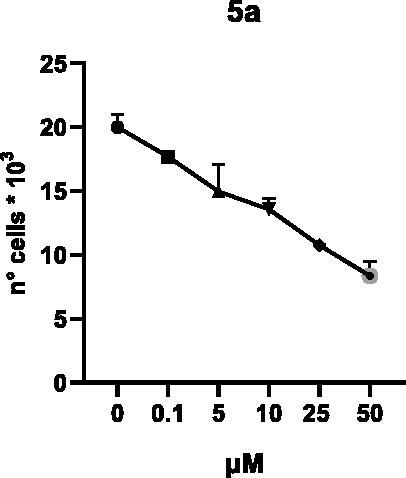	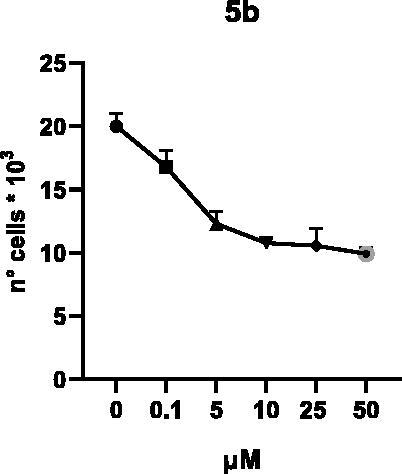	
**72 h**			
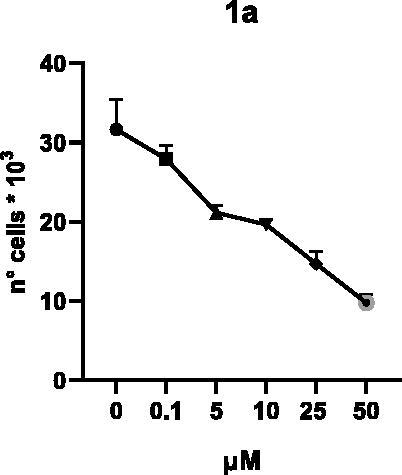	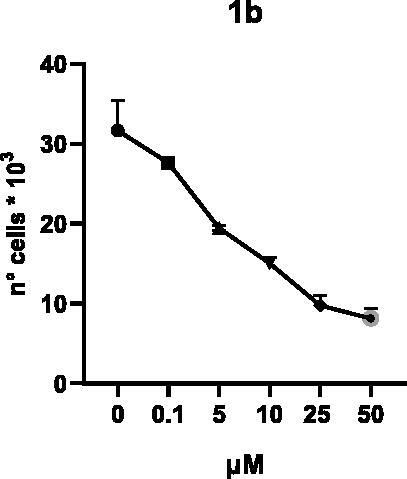	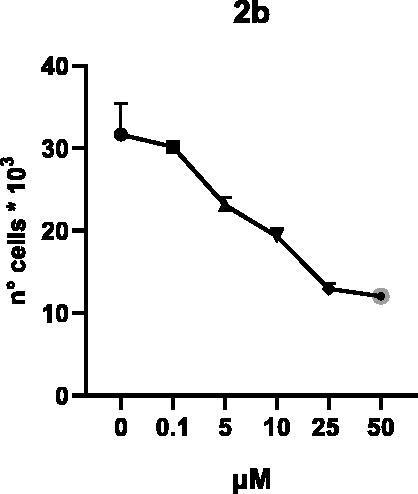	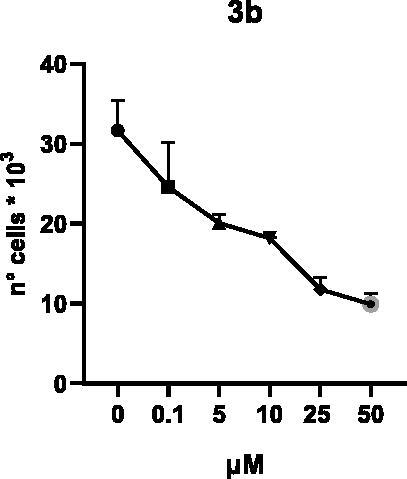
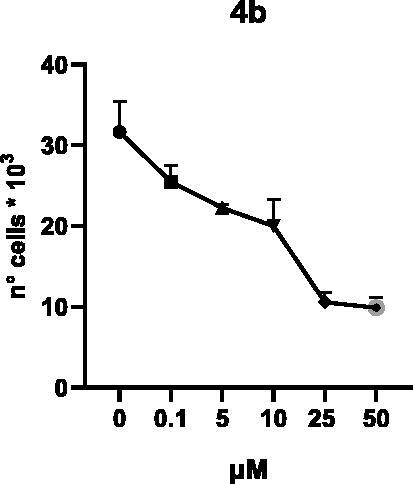	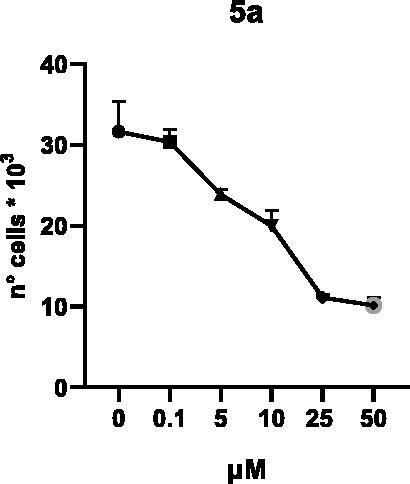	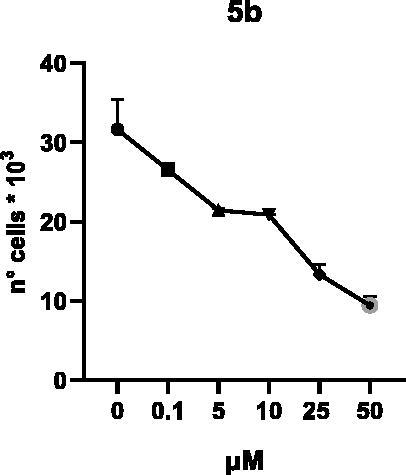	

In U937 cells the new LSD1 inhibitors showed moderate activities, with **1b**, **3b**, **5a**, and **5b** being the most potent and **1a**, **2b**, and **4b** almost ineffective at both time points. In this cell line, the *meta* phenyl and 3-thienyl derivatives **1b** and **5b** exerted the highest potency at 48 and 72 h. LNCaP cells were more sensitive than U937 cells to these inhibitors, likely due to the ability of the tested LSD1 inhibitors to also inhibit MAO-A, that is known to play a role in prostate cancer cell proliferation^30–33^. In LNCaP cells, all the tested compounds exhibited dose-dependent decreased cell viability. After 48 h of treatment **3b** was the most potent, with IC_50_ = 12.4 μM, and after 72 h all the tested inhibitors displayed IC_50_ values comprised between 9.9 and 25.4 μM, with **1b** and **3b** being the most effective (Table 6).

**Table 6.** IC_50_ values of **1a,b**, **2 b**, **3 b**, **4 b**, and **5a,b** in LNCaP cells. For comparison, the IC_50_ (72 h) of the selective MAO-A inhibitor clorgyline has been added.

Compd	LNCaP cells, IC_50_ (μM)	
	48 h	72 h
**1a**	34.1 ± 13.2	25.4 ± 3.2
**1b**	22.8 ± 5.1	9.9 ± 1.7
**2b**	27.6 ± 9.8	18.5 ± 2.7
**3b**	12.4 ± 6.1	13.4 ± 3.7
**4b**	25.7 ± 8.3	15.2 ± 3.5
**5a**	26.5 ± 4.8	17.9 ± 1.6
**5b**	32.1 ± 7.8	17.2 ± 3.5
clorgyline		63.7 ± 8.2^a^

^a^Ref. 30.

**Figure 5.**
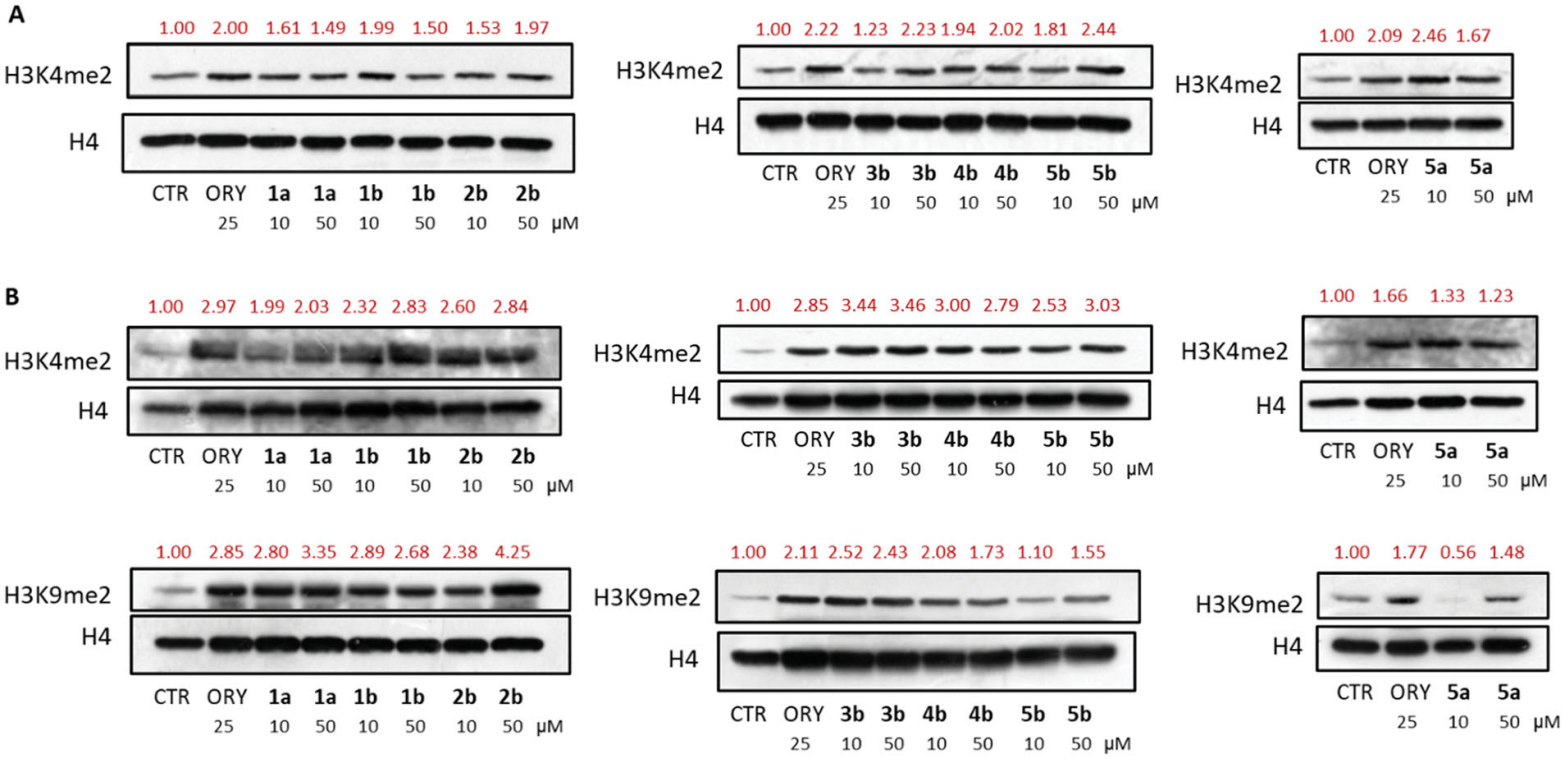
Western blot analyses of the H3K4me2 levels in U937 cells (**A**) and of H3K4me2 and H3K9me2 levels in LNCaP cells (**B**).

To link the reduction of cell viability to the inhibition of LSD1, western blot analyses have been performed on the new TCP-based inhibitors at 10 and 50 μM after 48 h of treatment to determine the changes in H3K4me2 (U937) or H3K4me2 and H3K9me2 (LNCaP) levels, typical marks for LSD1 demethylase activity (Figure 5). In LNCaP cells we also detected the levels of H3K9me2 because LSD1 is known to interact with the androgen receptor and to promote androgen-dependent transcription of target genes by ligand-induced demethylation of H3K9me1/2^4^^,^^34^. ORY-1001 (iadademstat)^35^, a known covalent LSD1 inhibitor, was used as a reference drug (at 25 μM).

Western blot analyses in both cell lines confirmed the capability of the tested compounds to inhibit LSD1 in cellular contexts, showing a moderate to huge increase of methylation levels of all the investigated marks.

## 4. Conclusions

The known anti-MAO drug TCP is the most reliable fragment to design potent, covalent LSD1 inhibitors, active in cancer cells as antiproliferative agents^16^. In our previous works, we identified MC2580^17^ and MC2584^18^ as TCP analogs with higher potency against LSD1. The insertion of a phenyl ring at the *para* position of the benzoylamino moiety of MC2584 led to **1a**, which retained potency against LSD1 and displayed high effects in MV4-11 and NB4 leukaemia cells^25^. Starting from these findings, we designed and prepared new **1a** analogs by shifting the phenyl ring from *para* to *meta* position (**1b**), and by inserting 5- or 6-membered heterocyclic rings either at *para* or *meta* positions (**2a,b**-**6a,b**). Compounds **1b**-**6** were tested against LSD1: the inhibition assay showed the *meta* derivatives (**b**) to be more potent than the corresponding *para* counterparts (**a**), with the *meta* 2- and 3-thienyl analogs **4b** and **5b** being the most potent (IC_50_ values = 0.015 (**4b**) and 0.005 (**5b**) μM). When tested against MAOs, **1b**-**6** were found active at submicromolar/nanomolar levels mainly against MAO-A. The comparison between the inhibitory activities of **1b**-**6** against the two families of flavoenzymes (LSD1 and MAOs) showed an LSD1-selective behaviour for all the *meta* regioisomers over MAO-B and in some cases respect to MAO-A, with the 2- and 3-thienyl derivatives **4b** and **5b** being the most LSD1-selective respect to MAO-B (selectivity indexes: 24.4 and 164, respectively).

Selected compounds **1a,b**, **2b**, **3b**, **4b**, and **5a,b** were tested in U937 AML and prostate cancer LNCaP cells to detect their antiproliferative activities after 48 and 72 h of treatment. LNCaP cells turned out to be more sensitive than U937 to these LSD1 inhibitors because all the tested compounds displayed a dose-dependent decrease of cell viability with **1b** and **3b** being the most effective. In U937 cells, only **1b**, **3b**, **5a**, and **5b** gave an effect, while the remaining compounds were less active or even inactive. Western blot analyses, performed in both the cell lines after 48 h of treatment with the same compounds to detect the levels of H3K4me2 (U937 and LNCaP) and H3K9me2 (LNCaP), confirmed the involvement of LSD1 inhibition in these cellular assays showing a general increase of the methylation levels.

Further studies will be performed with these compounds in different cancer contexts to assess their anticancer potential.
